# Polarization‐Sensitive Holotomography for Multidimensional Label‐Free Imaging and Characterization of Lipid Droplets in Cancer Cells

**DOI:** 10.1002/advs.202509420

**Published:** 2025-08-21

**Authors:** Hossein Khadem, Maria Mangini, Maria Antonietta Ferrara, Anna Chiara De Luca, Giuseppe Coppola

**Affiliations:** ^1^ Institute of Endotypes in Oncology, Metabolism and Immunology “Gaetano Salvatore” IEOMI Second Unit, National Research Council Via P. Castellino 111 Naples 80131 Italy; ^2^ Institute of Applied Sciences and Intelligent Systems “Eduardo Caianiello” ISASI Unit of Naples, National Research Council Via P. Castellino 111 Naples 80131 Italy

**Keywords:** birefringence imaging, cancer cell metabolism, lipid droplets, polarization‐sensitive holotomography, quantitative phase imaging

## Abstract

Lipid droplets (LDs) are key markers of cellular metabolism, often altered in cancer. While holotomography enables 3D, label‐free imaging of LDs via refractive index, it relies on complex thresholding and lacks biochemical specificity. Here, polarization‐sensitive holotomography (PS‐HT), which leverages the intrinsic birefringence of LDs for high‐contrast, selective identification with a fixed near‐zero threshold is presented. Using prostate cell models (healthy PNT2 and cancer PC3), PS‐HT is validated against fluorescence microscopy and holotomography, showing that it enables accurate quantification of birefringence, LD volume, dry mass, molecular organization, and spatial distribution. Cancer cells show significantly higher birefringence after glucose treatment, reflecting enhanced lipid accumulation. PS‐HT, combined with principal component analysis, achieves near‐perfect classification of cancer versus healthy cells, establishing it as a robust, label‐free tool for studying lipid metabolism and cancer diagnostics.

## Graphical abstract

Polarization‐sensitive holotomography (PS‐HT) enables label‐free 3D imaging and quantitative analysis of lipid droplets by exploiting their intrinsic birefringence. Applied to prostate cell models, PS‐HT reveals glucose‐induced lipid accumulation in cancer cells and achieves near‐perfect classification of healthy and cancerous states. This approach offers new possibilities for metabolic phenotyping and cancer diagnostics.

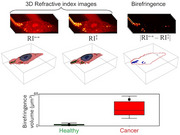

## 1 Introduction

Lipid droplets (LDs) are dynamic organelles that play a central role in cellular lipid metabolism and energy homeostasis.^[^ ^1^, ^2^ ^]^ Typically, lipid droplets exhibit a spherical morphology and range in diameter from a few hundred nanometers to several micrometers (commonly 0.5–5 µm), depending on the cell type, metabolic state, and external stimuli.^[^ ^3^ ^]^ Initially recognized as simple fat storage structures, they are now appreciated for their multifaceted roles in cell signaling, membrane synthesis, and protein trafficking.^[^ ^4^, ^5^ ^]^ In cancer biology, LDs have emerged as critical players in tumor development and progression, influencing key processes such as proliferation, metastasis, and resistance to therapy.^[^ ^6^, ^7^ ^]^ The physical properties of LDs—such as volume, mass, density, size, and morphology—provide vital insights into their functional roles and adaptive responses in cancer cells.^[^ ^8^ ^]^ For instance, LD size and density are closely associated with lipid metabolism reprogramming, a hallmark of cancer that supports rapid cell division and survival under metabolic stress. Moreover, changes in LD morphology and volume can serve as biomarkers for cancer cell states, reflecting alterations in lipid storage and utilization.^[^ ^9^ ^]^ Recent studies have also demonstrated that LDs can be used to discriminate cancer cells from healthy ones, as tumor cells tend to accumulate LDs due to the Warburg effect, where increased glucose (Glc) uptake is diverted toward lipid biosynthesis to support rapid proliferation and metabolic adaptation.^[^ ^10^, ^11^, ^12^ ^]^ These characteristics not only deepen our understanding of cancer biology but also provide promising opportunities for diagnostic tools and targeted therapeutic strategies.

Thanks to significant advancements in subcellular optical imaging, various aspects of LDs can now be studied in detail.^[^ ^13^ ^]^ Confocal fluorescence microscopy (CFM) allows visualization of LD distribution and dynamics and is widely used with lipid‐specific dyes such as BODIPY and Nile Red to stain lipid‐rich structures with high contrast.^[^ ^14^, ^15^ ^]^ However, CFM suffers from the need for labeling, which can interfere with cellular functionality and potentially alter lipid metabolism. Additionally, fluorescence imaging is limited in providing quantitative results, as fluorescence intensity depends on dye concentration, photobleaching, and variations in cellular uptake, leading to challenges in the precise quantification of LD content.^[^ ^16^ ^]^ Moreover, these dyes are not strictly specific for LDs, as they can also stain other neutral lipid‐rich structures, such as endoplasmic reticulum membranes or other intracellular membranes, further compromising the accuracy of LD identification.^[^ ^17^, ^18^ ^]^ As a label‐free technique, Raman microscopy can provide hyperspectral images of the biochemical structure distribution within LDs, allowing for the identification of specific lipid species and the quantification of lipid metabolism in living cells.^[^ ^19^, ^20^, ^21^ ^]^ However, the inherently weak Raman scattering necessitates longer integration times compared to CFM, often requiring several seconds to minutes per spectrum, which limits real‐time imaging capabilities. Quantitative phase imaging (QPI), another noninvasive and label‐free technique, provides high‐speed imaging of LDs by measuring optical phase shifts influenced by both cellular refractive index (RI) and thickness.^[^ ^22^, ^23^, ^24^, ^25^, ^26^, ^27^, ^28^ ^]^ Holotomography (HT), an advanced implementation of QPI, enhances this approach by utilizing optical diffraction tomography to reconstruct high‐resolution 3D maps of the subcellular *RI*.^[^ ^29^, ^30^, ^31^, ^32^ ^]^ HT operates by illuminating a sample with multiple angles of incident light and capturing the resulting diffraction patterns. These patterns are then computationally reconstructed using inverse scattering algorithms to generate a 3D *RI* distribution of the sample.^[^ ^33^ ^]^ This technique allows for the assessment of LD size, volume, density, *RI*, and dry mass, making it a valuable tool for studying lipid biophysical properties, metabolism, and cellular heterogeneity.^[^ ^34^, ^35^ ^]^ Despite these advancements, *RI*‐based segmentation remains a fundamental limitation. Since the *RI* of LDs can overlap with other high‐*RI* organelles, such as nucleoli, the approach still requires threshold‐based identification, which varies across cell types and biological conditions, reducing its reliability.^[^ ^36^, ^37^ ^]^ Additionally, while HT provides detailed 3D structural information and enables multi‐parameter analysis, it does not inherently differentiate LDs based on their biochemical composition, limiting its selectivity in distinguishing lipid‐rich structures from other dense organelles.^[^ ^38^, ^39^ ^]^ Recent advances in polarization‐sensitive tomographic imaging have demonstrated the potential of quantitative 3D birefringence mapping for biological samples, using high‐numerical‐aperture setups and polarized array sensors to simplify Jones matrix retrieval.^[^ ^40^ ^]^

In this study, we demonstrate that the addition of birefringence imaging through polarization‐sensitive holotomography (PS‐HT) enhances HT by providing an additional contrast mechanism for LD identification. First, we use fluorescence microscopy as a reference method to selectively discriminate LDs from other intracellular structures, ensuring accurate identification and validation of HT‐based segmentation. By correlating HT with fluorescence microscopy (a method with high selectivity for LDs), we determine the optimal *RI* thresholds (*RI*_th_) in two different cell lines with different treatments, enabling a more precise assessment of LDs. Structurally, LDs typically consist of a core of neutral lipids, primarily composed of triglycerides, cholesteryl esters, and fatty acids, surrounded by a phospholipid monolayer.^[^ ^41^ ^]^ The chain structure of these neutral lipids and their hydrophobic interactions contribute, unlike the cytosol, to creating an anisotropic packing that could explain the birefringence of LDs.^[^ ^42^ ^]^ Moreover, other subcellular organelles show minimal birefringence compared to LDs, enhancing the selectivity of their detection. By correlating birefringence measurements with fluorescence and HT imaging, we validate that birefringence originates exclusively from LDs, thereby linking physical and biochemical information. The high‐contrast visualization of LDs against a near‐zero birefringence background effectively eliminates the need for complex *RI*‐based thresholding. Indeed, birefringence intensity reflects the internal molecular organization and lipid anisotropy, while *RI* can be used to achieve information on LD volume and dry mass and the lipid density within individual LDs. Herein, these parameters are evaluated across two cell populations, specifically PNT2 (healthy prostate cells) and PC3 (prostate cancer cells), revealing significant metabolic differences between healthy and cancerous cells. Cancer cells, characterized by increased Glc uptake and lipid accumulation due to the Warburg effect, exhibit a markedly higher birefringence signal and larger LD volumes compared to their healthy counterparts. By leveraging these birefringence‐based features, we apply principal component analysis (PCA) and statistical classification methods to differentiate cancerous from healthy cells with ≈100% accuracy. These findings highlight the potential of PS‐HT as a rapid, label‐free, and high‐contrast imaging method for studying lipid metabolism and metabolic reprogramming in cancer, providing a promising tool for both fundamental research and clinical diagnostics.

## 2 Results and Discussion

### 2.1 Cell Selection

To investigate LD formation in a controlled biological system, we selected PNT2 (RRID: CVCL_2164) and PC‐3 (RRID: CVCL_0035) cell lines, which represent healthy and cancerous prostate cells, respectively. Both cell lines were obtained from a certified cell bank and regularly maintained under standard culture conditions (37 °C, 5% CO_2_, humidified atmosphere). To ensure the validity of the experimental results, all cell lines were routinely tested for mycoplasma contamination using a PCR‐based assay, and all results were consistently negative. These models were chosen due to their well‐documented metabolic differences: while PNT2 healthy cells maintain a balanced lipid metabolism with low LD levels, PC3 cancer cells undergo metabolic reprogramming, including increased lipid biosynthesis and storage as a result of the Warburg effect.^[^ ^43^, ^44^ ^]^ Consequently, cancer cells preferentially convert Glc into lactate even in the presence of oxygen, thus favoring aerobic glycolysis over mitochondrial oxidative phosphorylation. Indeed, prostate cancer cells, particularly aggressive variants such as PC3, have been shown to accumulate excess LDs as a survival strategy, utilizing them for energy storage, membrane synthesis, and protection against oxidative stress.^[^ ^45^ ^]^ The PNT2 and PC3 cell lines were exposed to Glc for 48 h to stimulate LD formation. Untreated cells, not exposed to Glc, served as controls. To ensure accurate identification of LDs and to distinguish them from other intracellular structures, we subsequently fluorescently labeled a subset of cells using BODIPY, a highly selective marker for LDs, and performed CFM (see Experimental Section, Supporting Information). The CFM images demonstrate the effects of Glc treatment on LD accumulation across the two cell models (Figure S1, Supporting Information). The images reveal a pronounced increase in LD number and size specifically in Glc‐treated PC3 cells. Given that LD accumulation correlates with tumor progression and serves as a potential biomarker, these cell lines provide an ideal model for comparing HT and PS‐HT in their ability to characterize lipid metabolism and discriminate cancerous from healthy cells. Specifically, we aim to demonstrate that birefringence imaging via PS‐HT enhances LD detection, offering improved contrast and selectivity over conventional *RI*‐based segmentation in HT.

### 2.2 HT, Fluorescence, and PS‐HT Microscopy

**Figure 1:**
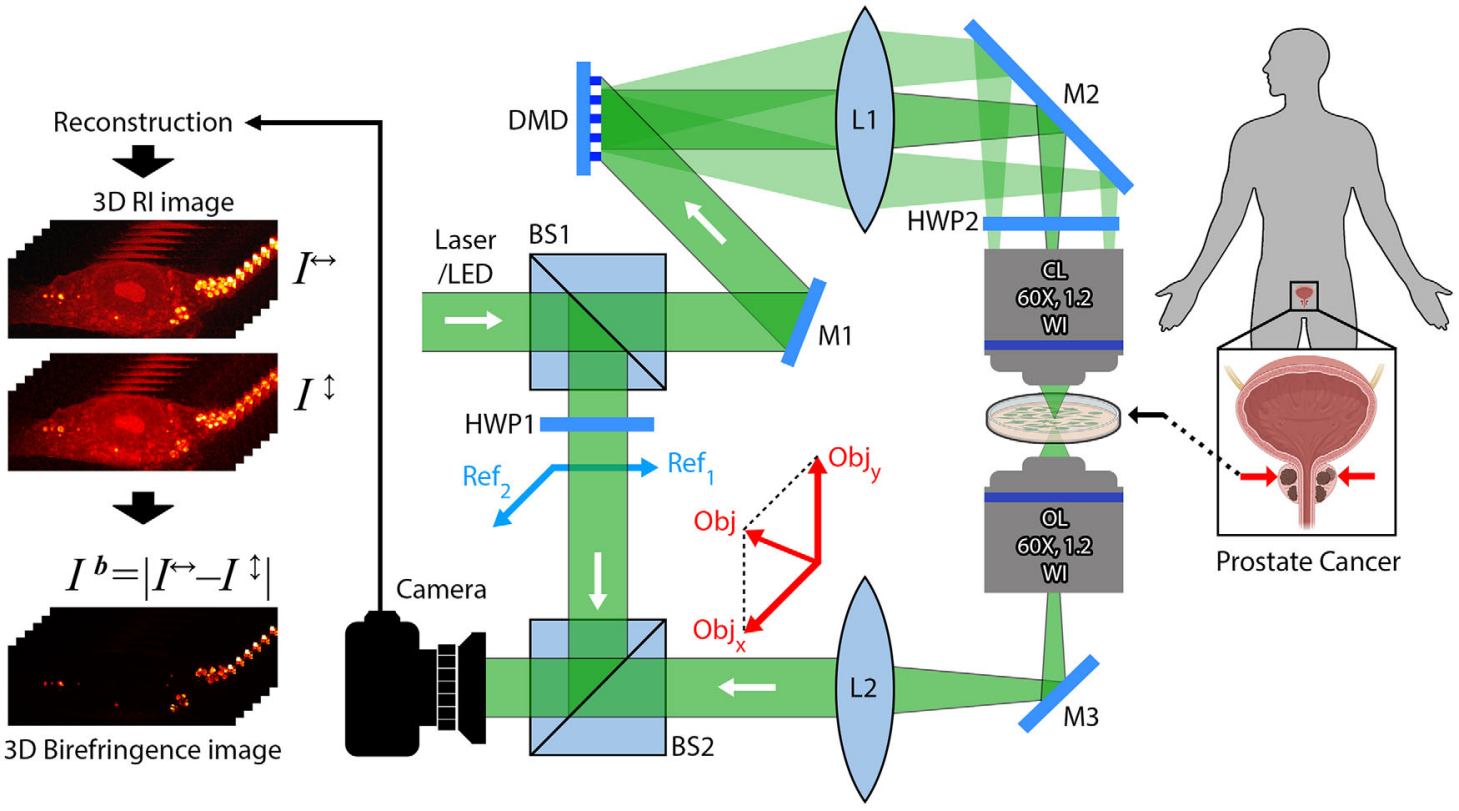
Multimodal optical setup for label‐free, 3D imaging of LDs. Schematic representation of the experimental setup integrating holotomography (HT), polarization‐sensitive holotomography (PS‐HT), and wide‐field fluorescence microscopy (WFM). In HT, a coherent laser beam is split into reference (Ref) and object (Obj) arms; the object beam illuminates the fixed sample at multiple angles via a digital micromirror device (DMD), while the resulting holograms formed by interference with the reference beam enable reconstruction of 3D refractive index (RI) maps. PS‐HT is achieved by introducing half‐wave plates (HWPs) in the reference arm and object arms, allowing acquisition of *RI* maps under two orthogonal polarization states. Birefringence images are calculated as the absolute difference between the two *RI* maps. For WFM, the laser is replaced by LED sources equipped with specific filters for excitation, and the reference arm is removed, enabling fluorescence‐based validation. Abbreviations: BS, beam splitter; L, lens; M, mirror; CL, condenser lens; OL, objective lens; WI, water immersion.

In this study, we employed a multimodal modified‐Tomocube microscope to acquire 3D *RI*, birefringence and wide‐field fluorescence microscopy (WFM) images of fixed cells (**Figure** **1**).^[^ ^46^ ^]^ In HT, the microscope is based on the optical diffraction tomography technique, where a laser beam is split into object and reference arms. The object beam interacts with the fixed cell at multiple illumination angles. The resulting forward‐scattered light interferes with the reference beam, forming a hologram on the camera plane for each illumination angle. By reconstructing these holograms, a high‐resolution 3D *RI* map of the sample can be retrieved in one single acquisition. To obtain birefringence images, we originally modified the Tomocube setup by inserting a half‐wave plate in the object arm (HWP2), to control the polarization state of the object beam, along with a motorized half‐wave plate in the reference arm (HWP1).^[^ ^47^, ^48^, ^49^, ^50^ ^]^ This configuration enables alternating hologram acquisition between two orthogonal polarization states (Figure S2, Supporting Information). These holograms are then processed computationally to retrieve the 3D *RI* maps of the sample for both orthogonal polarizations. By calculating the absolute difference between these *RI* maps, a 3D birefringence image is generated.^[^ ^51^ ^]^ This approach allows for the volumetric mapping of birefringence within the sample, where LDs can be distinguished based on their high optical anisotropy. For WFM, the laser source is replaced by an LED excitation source with three filter sets (385, 475, and 570 nm) that illuminate only the object arm. A normal incidence angle is used for illumination, and the sample is scanned in a point‐wise manner. WFM images are acquired under these conditions to provide biochemical validation. Throughout this study, this multimodal microscopy system was employed to image PNT2 and PC3 cells under various Glc treatment conditions for comprehensive analysis and characterization.

**Figure 2:**
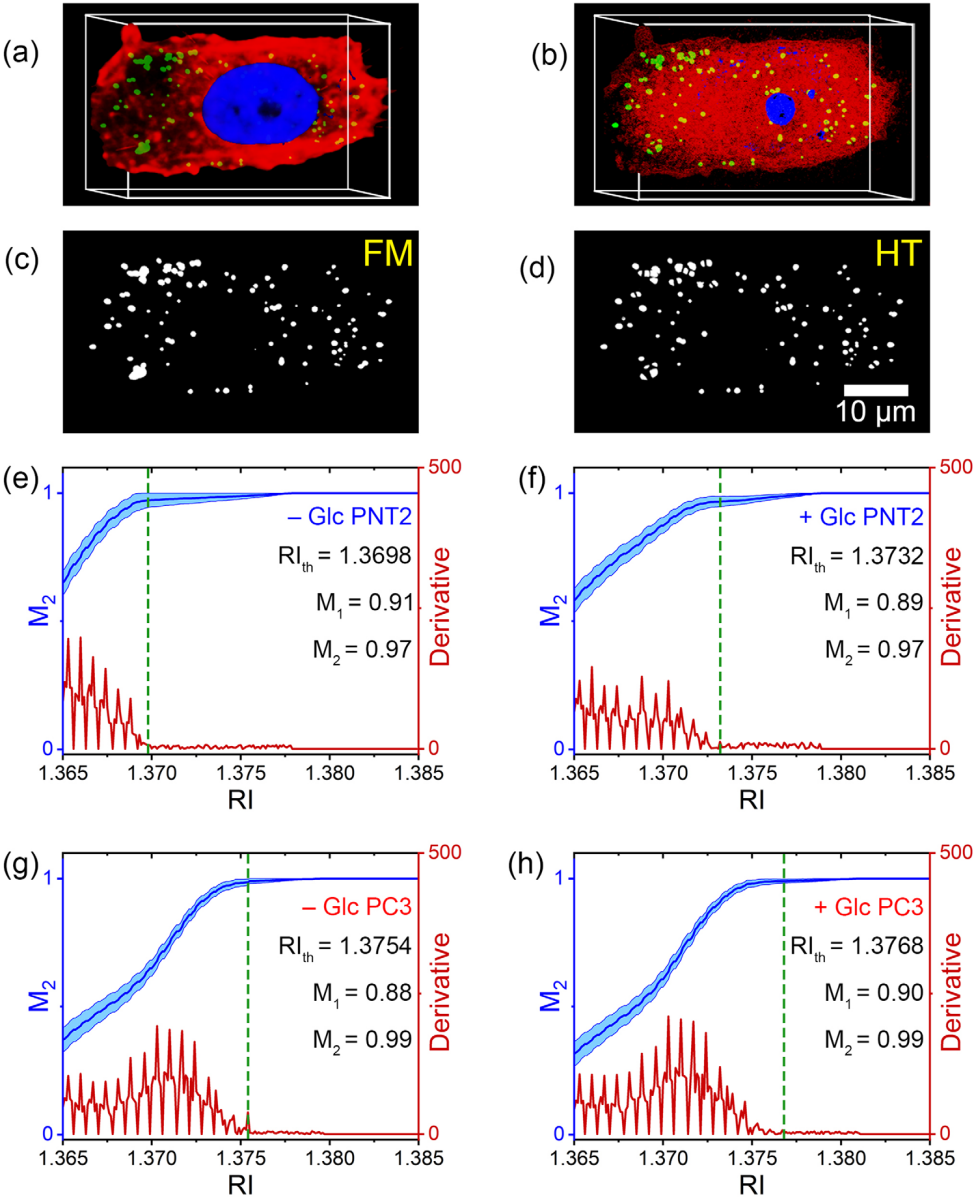
Correlative fluorescence and holotomography imaging for LD segmentation and *RI* threshold optimization. 3D imaging of a Glc‐treated PC3 cell acquired using a) WFM and b) HT. LDs are visualized in green, the cytosol in red, and the nucleus in blue. Corresponding binary maximum intensity projection (MIP) images of LDs (green) segmented from c) WFM and d) HT highlight the spatial distribution of LDs in both modalities. e–h) Optimization of refractive index threshold for LD segmentation using colocalization analysis in four cell populations: e) untreated PNT2, f) Glc‐treated PNT2, g) untreated PC3, and h) Glc‐treated PC3. In each panel, the blue curve represents the *M*_2_ colocalization coefficient as a function of *RI*, while the red curve shows its first derivative. The vertical green dashed line marks the optimal *RI* threshold (*RI*_th_), defined as the smallest *RI*‐value at the onset of the *M*_2_ plateau. Each panel includes the calculated *RI*_th_, along with *M*_1_ and *M*_2_ values at *RI*_th_.

**Figure** **2**a shows a 3D WFM image of a PC3 cell incubated with Glc for 48 hours, visualized in red (F‐actin), green (lipids), and blue (nucleus) channels. Although BODIPY exhibits high selectivity for staining lipids, lipid‐rich domains such as the endoplasmic reticulum and other intracellular cell membranes can contribute to the fluorescence signal, making it inaccurate to assume that the signal originates solely from LDs.^[^ ^52^ ^]^ Therefore, in addition to fluorescence intensity, the morphology and spatial localization of vesicles within specific regions of interest (ROIs) were also considered. Figure 2b presents the corresponding HT image acquired from the same optical field of view (see also Movie S1, Supporting Information). To identify subcellular components, we applied *RI* thresholds based on values summarized in Table S1 (Supporting Information). Generally, the cytosol exhibits lower *RI*‐values (1.335 ≲ *RI* ≲ 1.350), nuclei show intermediate *RI* (1.35 ≲ *RI* ≲ 1.365), and LDs display significantly higher *RI‐*values (*RI* ≳ 1.375).^[^ ^53^, ^54^ ^]^ For visual comparison with WFM, the segmented HT regions are color‐coded as red (cytosol), blue (nucleus), and green (LDs). To quantify colocalization and identify the optimal *RI* threshold (*RI*_th_) for each cell line, binary maximum intensity projection (MIP) of the green colored images (LDs) from both WFM and HT were generated (Figure 2c,d). MIP consolidates all 3D LD features into a single frame while minimizing the axial blur in WFM caused by its non‐confocal nature. Using the WFM MIP image as the reference, we swept the *RI* threshold from 1.3650 to 1.3850 in steps of 0.0001, calculating colocalization coefficients *M*_1_ (i.e., the fraction of fluorescently labeled LDs in the WFM MIP image that overlap with LDs identified in the HT MIP image) and *M*_2_ (i.e., the fraction of LDs identified in the HT MIP image that overlap with fluorescently labeled LDs in the WFM MIP image) at each step. The smallest *RI* threshold corresponding to the plateau of maximum *M*_2_ values was selected as the optimal threshold (*RI*_th_). Figure 2e–h illustrates the trend of increasing colocalization between WFM and HT images as a function of *RI*. To enhance visualization of this trend, the derivative of the *M*_2_ colocalization coefficient with respect to *RI* is also plotted. The green dashed line indicates the lowest *RI*‐value within the colocalization plateau, which is defined as the optimal *RI* threshold (*RI*_th_) for accurate LD segmentation in HT. Beyond this threshold, further increases in *RI* result in negligible changes in *M*_2_ and its derivative. Generally, at the *RI*_th_, the *M*_2_ coefficient is greater than *M*_1_. This suggests that LDs appear larger in WFM images compared to their counterparts in HT, likely due to structural optical differences between the two imaging modalities, such as variations in point spread functions and optical resolution (Figure S3, Supporting Information). Nevertheless, both *M*_1_ and *M*_2_ values at *RI*_th_ are typically above 0.9, indicating a high degree of agreement between the two imaging techniques. Notably, *RI*_th_ varies across different cell lines and treatment conditions, indicating the lack of a universal *RI* threshold for LD identification. This variability emphasizes the limitations of *RI*‐based segmentation, as threshold selection is highly dependent on the cellular context. Fluorescence labeling has a negligible effect on the *RI* of LDs, as confirmed in Figure S4 (Supporting Information). Indeed, the *RI* distribution of LDs in PNT2 and PC3 cells—under both unlabeled and BODIPY‐labeled conditions, with and without Glc treatment—reveals only minimal differences that do not affect the overall interpretation. Therefore, the *RI*_th_ obtained from labeled cells can be reliably applied to unlabeled cells. Accordingly, all subsequent analyses in this study were conducted using unlabeled cells.

**Figure 3:**
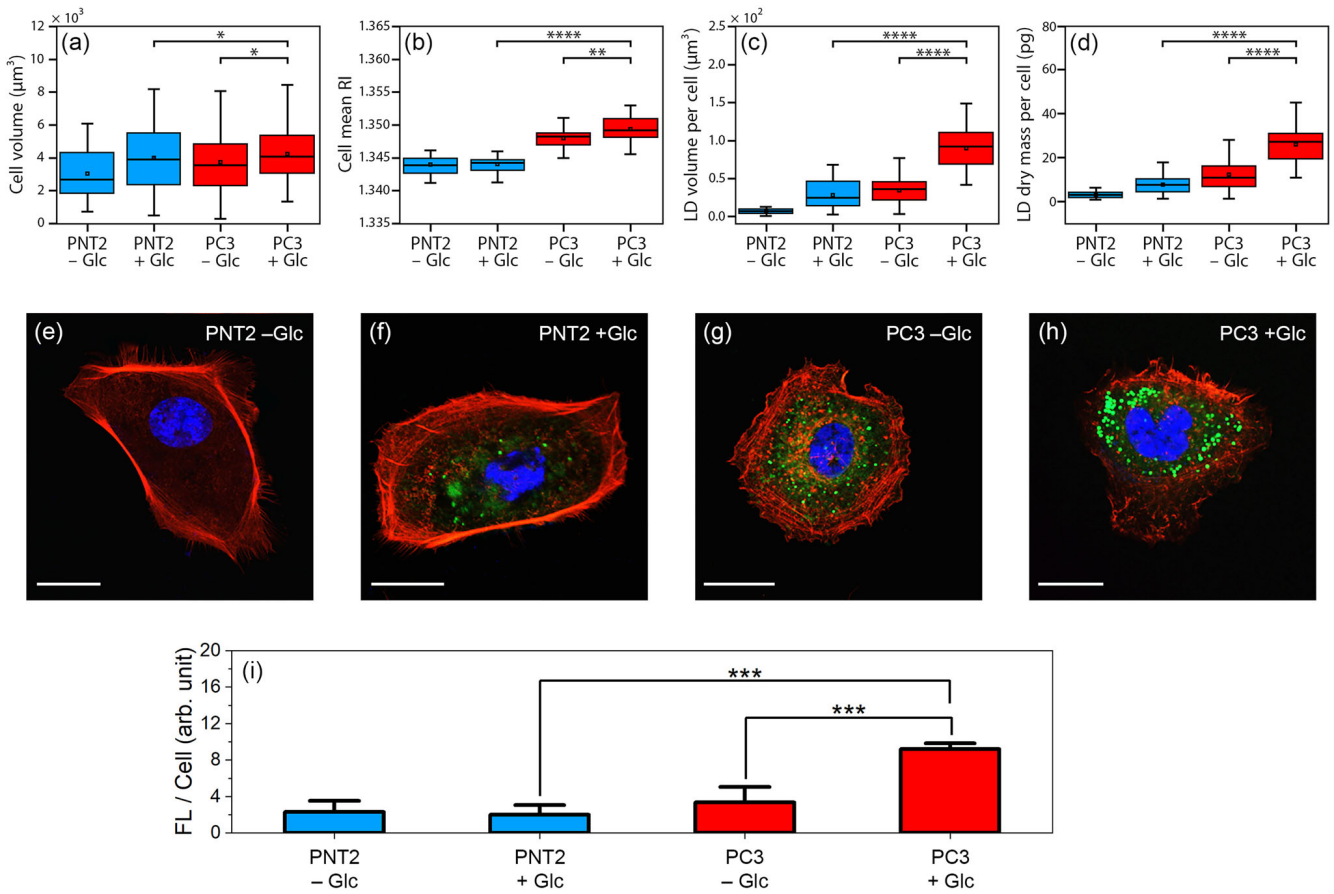
Glc‐induced modulation of cellular biophysical parameters measured by HT and CFM. a–d) Quantitative analysis of biophysical parameters extracted from 3D *RI* maps acquired via HT: a) whole‐cell volume, b) mean cellular *RI*, c) total LD volume per cell, and d) total LD dry mass per cell. e–h) Representative CFM images of PNT2 and PC3 cells under untreated (−Glc) and Glc‐treated (+Glc) conditions. Each panel shows merged channels (red: F‐actin, green: LDs, blue: nuclei); scale bars: 20 µm. i) Quantitative analysis of LD formation expressed as average green fluorescence intensity per cell (FL/Cell). Statistical significance was assessed using two‐sided Wilcoxon rank‐sum tests. *p < 0.05, **p < 0.01, ***p < 0.001, ****p < 0.0001.

Having determined RIth for LDs in each cell line, key biophysical properties such as LD volume and mass can be quantified, enabling the monitoring of LD formation and accumulation induced by Glc treatment. Since light polarization has minimal impact on the quantitative results, we arbitrarily selected the vertical polarization map for computing morpho‐physical parameters such as dry mass and density using HT (Figure S5, Supporting Information). Quantitative measurements—including cell volume, mean cellular *RI*, LD volume per cell, and LD dry mass per cell—show that cell volume remains nearly constant across all cell lines (**Figure** **3**a). This uniformity ensures a fair comparison of LD volume under different conditions. Regarding the mean *RI* of the entire cell, it is expected that the formation and accumulation of LDs would have a minimal impact on altering the mean *RI*, given that the volume of LDs is significantly smaller than the total cell volume. However, HT analysis revealed that PC3 cancer cells exhibit a higher mean *RI* compared to PNT2 healthy cells (Figure 3b). Furthermore, Glc treatment appears to induce changes in the mean *RI* of cancer cells, while in healthy cells, the mean *RI* remains nearly identical between treated and untreated conditions. These results can be attributed to a significant increase in LD formation, driven by the altered metabolic state of tumor cells (Warburg effect). This metabolic reprogramming favors the de novo synthesis of fatty acids, which are subsequently activated by fatty acyl‐CoA synthetases. Once activated, these fatty acids are esterified into triacylglycerols or sterol esters and predominantly stored within lipid droplets, reflecting their critical role in supporting the biosynthetic and energetic demands of proliferating cancer cells.^[^ ^55^ ^]^

From the quantitative analysis of LD volume in Figure 3c, it is evident that total LD volume is greater in Glc‐treated cells compared to untreated ones for both PNT2 and PC3. However, this increase is markedly more pronounced in cancer cells. Glc‐treated PC3 cells exhibit approximately three times greater LD volume compared to Glc‐treated PNT2 cells. This highlights the fundamentally different metabolism between healthy and cancer cells. This increase in volume is accompanied by a rise in LD dry mass, indicating greater accumulation of cholesteryl esters and fatty acids within LDs (Figure 3d).^[^ ^56^, ^57^ ^]^ To validate the quantitative LD measurements obtained through HT, we utilized CFM with BODIPY staining as a biochemical reference. Figure 3e–h shows representative confocal microscopy images of PNT2 (Figure 3e,f) and PC3 (Figure 3g,h) cells cultured in glucose‐deprived (−Glc) and glucose‐enriched (+Glc) conditions. All samples were stained with DAPI (blue) to visualize the nuclei, phalloidin (red) to label filamentous actin, and BODIPY (green) to detect LDs. Under Glc deprivation (Figure 3e), PNT2 non‐tumoral epithelial cells exhibited well‐organized F‐actin stress fibers and negligible BODIPY signal, indicating low lipid content. Upon Glc supplementation (Figure 3f), PNT2 cells showed a notable increase in green fluorescence. Interestingly, BODIPY staining appeared not only as small distinct micro‐spheres, typically associated with LDs, but also as a diffuse cytoplasmic signal. This diffuse pattern may reflect the accumulation of other neutral lipid species or altered membrane lipid composition induced by Glc exposure. It also highlights a key limitation of BODIPY: although widely used to label LDs, it lacks absolute specificity and can also associate with other lipid‐rich compartments, potentially confounding interpretation in metabolically active conditions.^[^ ^58^ ^]^ While moderate BODIPY staining was detected in Glc‐deprived PC3 cells, Glc reintroduction markedly increased the BODIPY signal, indicative of enhanced lipid storage (Figure 3g,h). The actin cytoskeleton appeared fragmented and less organized compared to PNT2 cells, in line with the increased plasticity typically seen in malignant cells undergoing metabolic adaptation. Although confocal microscopy enables high‐resolution, multiplexed imaging of subcellular structures, a major technical limitation remains the acquisition time. Each image in Figure 3 required ≈90 s to capture due to sequential scanning across multiple fluorescence channels. This time‐consuming process limits throughput and increases the risk of photobleaching and phototoxicity, particularly in live‐cell imaging or when using sensitive fluorophores. Nevertheless, confocal imaging remains a valuable technique for detailed morpho‐functional analyses, especially when combined with appropriate controls and complementary assays to validate staining specificity. Importantly, Figure 3i quantifies this trend by presenting the average BODIPY fluorescence intensity per cell, revealing a significant increase in LD‐associated fluorescence signal in Glc‐treated PC3 cells compared to all other conditions. This elevated fluorescence signal correlates strongly with the HT‐derived metrics of LD volume and dry mass (Figure 3c,d), providing cross‐validation between biochemical staining and label‐free imaging. The parallel increase in both fluorescence signal and *RI‐*based measurements confirms that HT accurately captures the enhanced lipid accumulation induced by metabolic reprogramming in cancer cells.

Although the strong correlation between HT and fluorescence microscopy results (Figure 3; Figure S3, Supporting Information) underscores the strength of HT as a label‐free, fast, and quantitative imaging method, the lack of universal *RI* thresholds introduces complexity to this approach. Furthermore, the non‐selectivity of *RI* in distinguishing subcellular compartments, including LDs, necessitates supervised analysis and definition of complex regions of interest to ensure accurate segmentation. For example, in certain conditions, the *RI*‐values of nucleoli may resemble those of LDs, making them challenging to differentiate during the thresholding process (Figure S6, Supporting Information). Although defining regions of interest can effectively separate nucleoli from LDs due to their spatial separation, this approach complicates and prolongs the analysis process for LD characterization in HT.

### 2.3 Volumetric Birefringence Imaging of LDs Using PS‐HT

**Figure 4:**
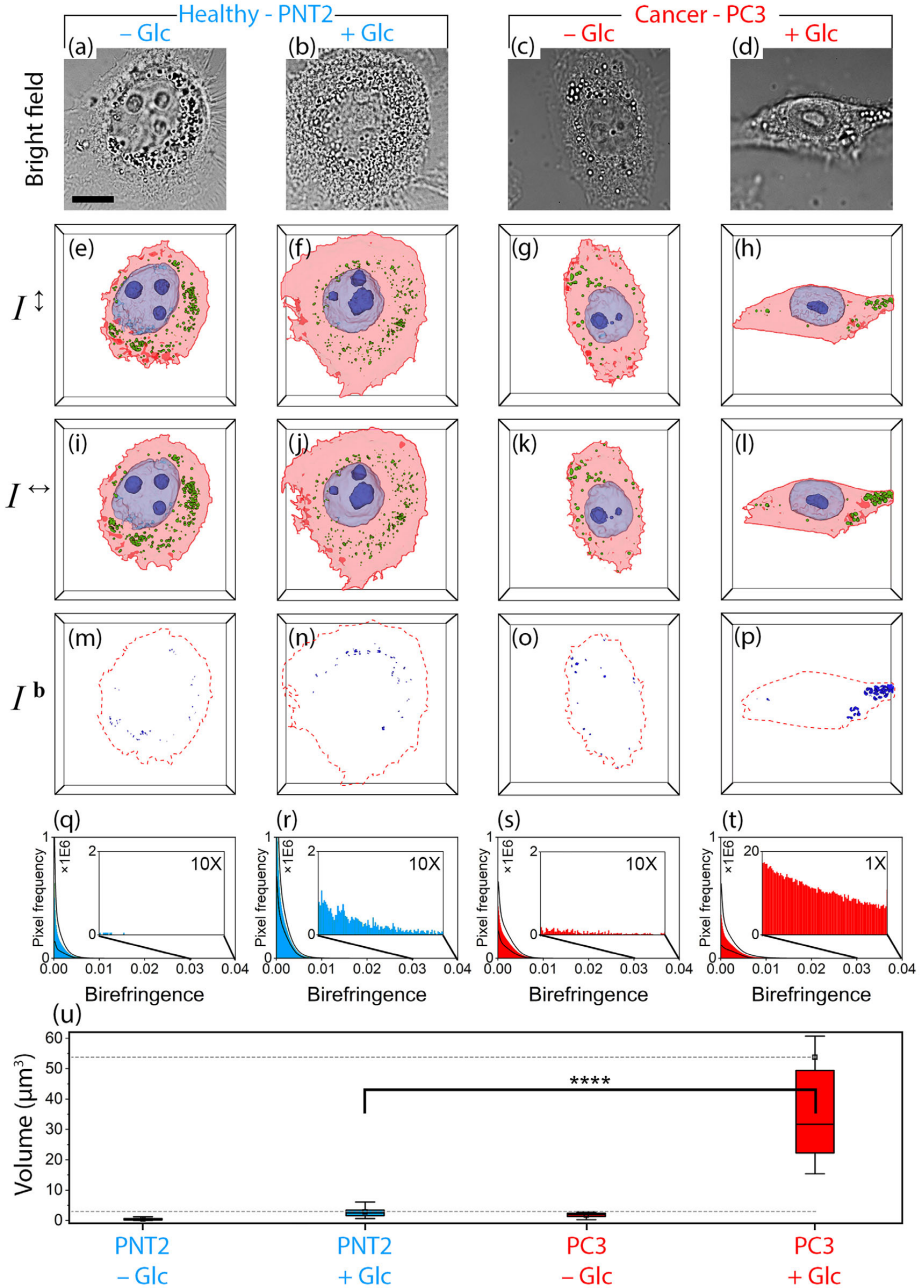
Volumetric birefringence imaging reveals Glc‐induced lipid droplet accumulation in prostate cancer cells. Images of PNT2 and PC3 cells in untreated (−Glc) and Glc‐treated (+Glc) conditions: a–d) Bright‐field images, e–h) 3D HT images for vertical polarization, i–l) 3D HT images for horizontal polarization, m–p) 3D birefringence images, q–t) Birefringence histograms of 3D images, and u) Total volume of intracellular regions with birefringence greater than 0.01. The scale bar (applies to all images) and the image depth for 3D images are 10 µm. Statistical significance was assessed using two‐sided Wilcoxon rank‐sum tests: ****p < 0.0001. See Table S1 (Supporting Information) for the thresholds used for segmenting each organelle in each cell line.

**Figure** **4** illustrates the use of PS‐HT to visualize and quantify LDs in three dimensions across different cell types. The goal is to exploit the birefringent properties of LDs to distinguish them from other subcellular structures and to assess differences in LD content between healthy and cancerous cells. In bright‐field images, LDs appear as circular vesicles, characterized by their bright appearance due to their high optical transmission in the visible range (Figure 4a–d).^[^ ^59^ ^]^ However, bright‐field microscopy does not allow a reliable distinction of LDs from other organelles, as it lacks molecular or structural specificity. By reconstructing *RI* images from two orthogonal polarizations, 3D birefringence images can be reconstructed using the following equation:

(1) $$I^{b}=\left|I^{↕}\;-\;I^{↔}\right|\;$$

where *I^b^* represents the 3D birefringence image, while​ *I* ^↕^ and *I* ^↔^ correspond to the 3D *RI* images obtained using vertical and horizontal polarizations, respectively. The horizontal and vertical polarization components used to obtain *I* ^↕^​ and ​*I* ^↔^ are defined within a frame that determines the orientation of the object beam's polarization vector relative to that of the reference beam.^[^ ^60^ ^]^ A comparison between *I* ^↕^ and *I* ^↔^ reveals subtle differences in the cytosolic boundary regions, which may result from the similar *RI* to the aqueous background and the thin structure of these regions (Figure 4e–l). The 3D birefringence images, generated using Equation (1), clearly show the anisotropic optical behavior of the lipid droplets. By applying a fixed near‐zero threshold (0.01 in this case) across all cell lines, birefringence segmentation can be effectively performed for visualization purposes (Figure 4m–p). A comparison with HT images confirms that this birefringence signal originates exclusively from LDs (Movie S2, Supporting Information). This eliminates not only the need for defining regions of interest for LD identification but also the variability in threshold selection, as unlike *RI* images, the threshold can remain constant and close to zero, simplifying the LD identification process. Due to their anisotropic molecular structure, resulting from a phospholipid monolayer and a cholesteryl ester‐rich core, LDs exhibit high sensitivity to light polarization.^[^ ^61^ ^]^ This structural anisotropy leads to significantly higher birefringence compared to other subcellular components, which predominantly have isotropic structures. Moreover, as observed, Glc‐treated PC3 cells exhibit increased birefringence, reflecting their enhanced LD accumulation. In contrast, while healthy cells also contain LDs, as observed in HT images, their low Glc uptake and limited accumulation of lipids and cholesteryl esters result in significantly reduced birefringence compared to Glc‐treated cancer cells.

By analyzing the birefringence histograms, it is evident that the pixel frequency in non‐birefringent regions (<0.01) exhibits a prominent peak across all cell lines, primarily corresponding to the total non‐birefringent volume of the cytosol, nucleus, and nucleoli (Figure 4q–t). Considering Glc‐treated cells, this peak is approximately twice as large in PNT2 cells compared to PC3 cells. Since the total cell volume remains nearly identical across all cell lines (Figure 3a), the reduced non‐birefringent volume in PC3 cells suggests a redistribution of volume toward birefringent regions. As birefringence exceeds 0.01, the pixel frequency decreases exponentially. Glc‐treated PC3 cells exhibit significantly higher birefringence compared to untreated PC3 and both Glc‐treated and untreated PNT2, particularly in regions with high birefringence values. For birefringence values greater than 0.03, the pixel frequency in Glc‐treated PC3 cells is ≈20 times higher than that of their healthy counterpart, PNT2 (Figure 4r,t). The observed results indicate that PC3 cancer cells exhibit distinct metabolic pathways for Glc uptake and lipid synthesis, leading to significant birefringence differences between cancerous and healthy cells.

Volumetric analysis (see Experimental Section, Supporting Information) of regions with birefringence greater than 0.01 reveals that the birefringent volume in Glc‐treated PC3 cells is ≈17 times larger than that in Glc‐treated PNT2 cells (Figure 4u). As previously mentioned, quantitative HT analysis indicates that the LD volume in Glc‐treated PC3 cells is approximately three times greater than that in Glc‐treated PNT2 cells (Figure 3c). Given that the volumetric birefringence measurements reveal a ≈17‐fold difference between the same conditions, we estimate that birefringence imaging by PS‐HT provides approximately sixfold higher sensitivity than conventional HT in detecting cancer‐induced metabolic alterations (Figure 3c and Figure 4u). While this analysis, similar to *RI* images, requires thresholding, the use of a fixed near‐zero threshold enables a more straightforward and universally applicable identification compared to *RI*‐based segmentation. Additionally, the birefringent volume in cancer cells demonstrates significantly greater sensitivity to Glc uptake—approximately six times more—than their *RI*‐based volume. By applying PCA to birefringence images, cancer cells can be distinguished from healthy cells with ≈100% accuracy, without the need for thresholding (Figure S7, Supporting Information).

**Figure 5:**
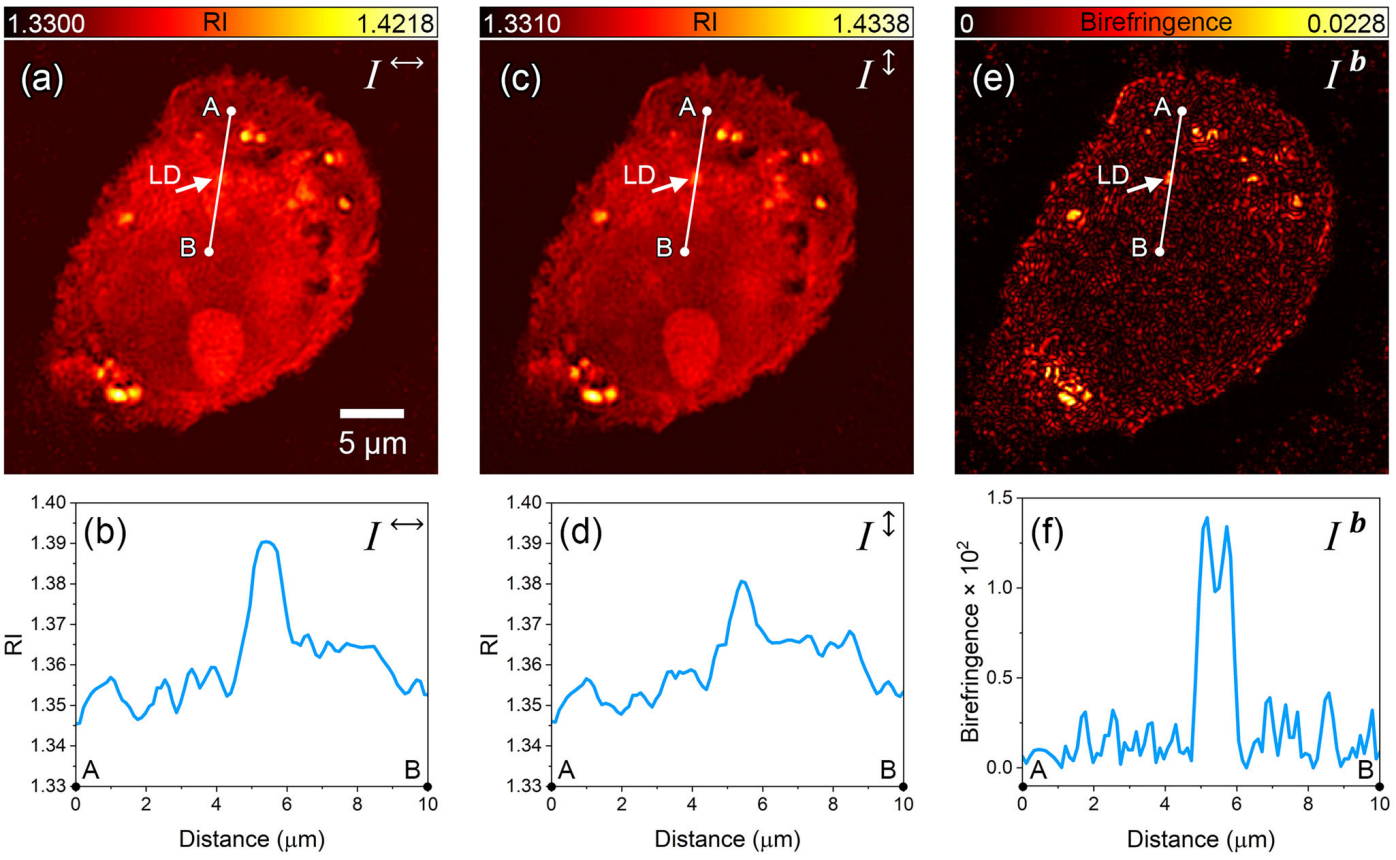
Contrast enhancement and internal structural profiling of LDs using birefringence imaging. Linear profiles from point A to B across an LD in a Glc‐treated PC3 cell at slice 110 out of 210 in the 3D image stack obtained under a,b) horizontal, c,d) vertical polarizations, and e,f) the birefringence image.

A comparative analysis of PS‐HT and HT images provides deeper insight into the advantages of birefringence imaging over *RI* imaging. **Figure** **5** illustrates the linear profiles of *RI* and birefringence for an LD within a 3D reconstructed slice of the *RI* and birefringence images, highlighting differences between the two modalities. As observed in the *RI* profiles (Figure 5b,d), the cellular background exhibits complex behavior with irregular fluctuations, complicating the selection of a universal threshold for detecting LDs. In contrast, in the birefringence image, the cellular background remains nearly constant and close to zero, allowing LDs to be observed with significantly higher contrast compared to *RI* images (Figure 5e,f). Additionally, while LDs appear as a single peak in the *RI* profile, they manifest as a double peak in the birefringence profile (Figure 5b,d,f). This structural differentiation reflects the internal heterogeneity of LDs, distinguishing the core from the surrounding shell. This phenomenon can be attributed to the highly ordered phospholipid monolayer surrounding LDs, which exhibits strong birefringence due to its radially oriented molecular arrangement. Furthermore, the anchoring force between cholesteryl esters and phospholipids at the core–shell interface of LDs leads to a more ordered radial orientation of cholesteryl esters near the surface compared to the core.^[^ ^62^ ^]^ As a result, the peripheral regions of LDs exhibit higher birefringence than the central regions. Therefore, PS‐HT imaging can provide more structural information compared to HT.

**Figure 6:**
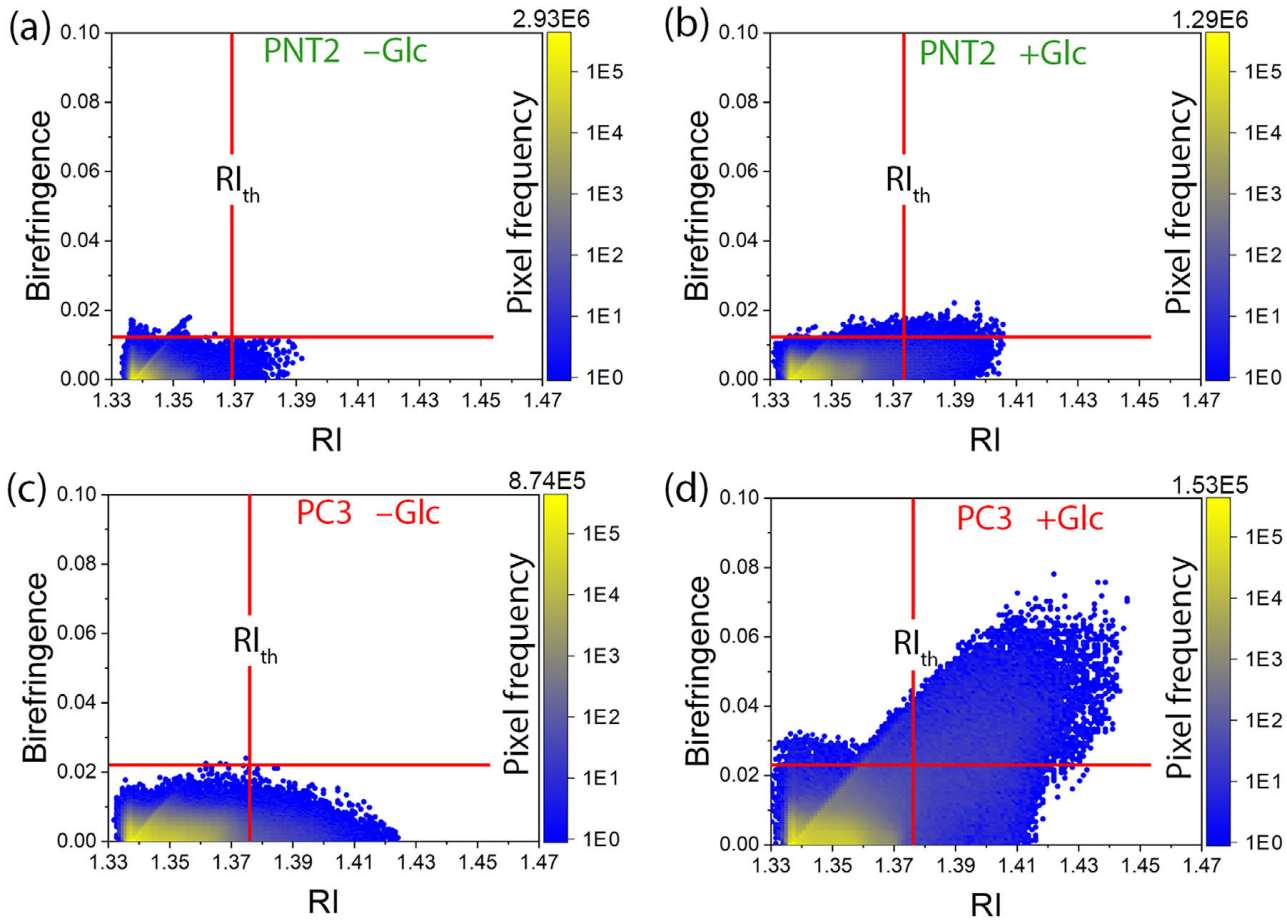
Selective association of birefringence with LDs as identified by *RI*_th_. Colocalization plots of pixel‐wise birefringence versus *RI* for four experimental conditions: untreated and Glc‐treated PNT2 (a,b), and untreated and Glc‐treated PC3 (c,d). Each pixel is color‐coded by its frequency, as indicated by the adjacent color bar. The vertical red line in each panel represents the *RI* threshold (*RI*_th_) used to identify LDs, as previously determined via colocalization between HT and WFM images (see Figure 2). Pixels with *RI* above this threshold (*RI* > *RI*_th_) are considered representative of LDs. The horizontal red line marks the maximum birefringence observed in the untreated condition for pixels with *RI* > *RI*_th_, and it serves as a reference for detecting birefringence enhancement following Glc treatment. For clarity, this horizontal line is overlaid on both treated and untreated plots of each cell line.

Colocalization analysis between *RI* and birefringence images supports the hypothesis that the higher birefringence observed in cancer cells, compared to healthy cells, is induced by Glc uptake and originates exclusively from LDs (**Figure** **6**). In the colocalization plots, the *RI*_th_ determined for LDs in each cell line is indicated by a vertical line. Additionally, the maximum birefringence observed in the untreated state of a given cell line is marked by a horizontal line. For comparative purposes, this horizontal line is also overlaid on the colocalization plot of the Glc‐treated condition. The colocalization plot of untreated PNT2 cells (Figure 6a) reveals that the birefringence in the LD region (*RI* >* RI*_th_) is not significantly different from that in the lower *RI* region (*RI* <* RI*_th_). Upon Glc treatment, the *RI* distribution shifts toward higher values (Figure 6b). Notably, birefringence remains largely unchanged in the *RI* <* RI*_th_ region but increases in the LD region following Glc treatment. The lack of birefringence changes in the *RI* <* RI*_th_ region, coupled with its increase in the LD region after Glc treatment, suggests that the birefringence enhancement is exclusively attributed to the formation and accumulation of LDs induced by Glc treatment. For PC3 cells, the trends in birefringence changes are similar to those in PNT2 cells. Glc treatment dramatically increases the birefringence in the LD region (*RI* > *RI*_th_) by nearly fourfold, while the birefringence in the *RI* < *RI*_th_ region remains largely unchanged (Figure 6c,d). Since the increase in birefringence in both cell lines occurs exclusively in the *RI* region corresponding to LDs (*RI* > *RI*_th_), it can be concluded that birefringence enhancement induced by Glc treatment is solely attributable to LD formation and accumulation, which is driven by Glc uptake. This observation highlights the distinct metabolic characteristics of cancer cells in Glc uptake and lipid synthesis.

## 3 Conclusion

In this study, we introduce PS‐HT as an advanced label‐free imaging modality capable of selective identification and detailed characterization of LDs within cellular environments. By exploiting the intrinsic birefringent properties of LDs, PS‐HT offers a high‐contrast visualization against a near‐zero birefringence background, thus overcoming key limitations of conventional HT, such as the need for complex, cell‐type‐specific *RI* thresholding. Through a multimodal validation approach combining PS‐HT, fluorescence microscopy, and conventional HT, we have confirmed that the detected birefringence signals originate exclusively from LDs. This validation facilitates reliable and consistent segmentation of LDs under varied biological conditions using a universal, fixed threshold. The pronounced optical anisotropy of LDs, driven by their neutral lipid core (primarily composed of triglycerides, cholesteryl esters, and fatty acids) surrounded by ordered phospholipid monolayers, produces strong birefringence signals, distinctly differentiating them from other subcellular structures.

PS‐HT generates comprehensive and multidimensional datasets for LD analysis, enabling simultaneous extraction of multiple critical parameters, including morphological characteristics (size, shape, and 3D spatial distribution), biophysical attributes (refractive index, birefringence intensity, dry mass, and density), structural details (shell–core organization inferred from birefringence profiles), and metabolic indicators (levels of lipid accumulation and molecular ordering alterations associated with metabolic reprogramming). Collectively, these capabilities allow the detailed examination of both quantitative and qualitative LD features at sub‐organelle resolution, thus providing a holistic phenotypic fingerprint of these crucial lipid storage organelles. Applying PS‐HT to prostate cell models revealed significant disparities in LD properties between healthy (PNT2) and cancerous (PC3) cells. Notably, Glc‐treated PC3 cancer cells exhibited substantially increased birefringence signals and enlarged LD volumes, indicative of augmented lipid accumulation and enhanced structural anisotropy—distinctive hallmarks linked to cancer metabolism, including the Warburg effect and upregulated de novo lipogenesis. The sensitive detection of these subtle, yet biologically relevant, differences underscores the strong potential of PS‐HT as a diagnostic tool for metabolic alterations in cancer.

In comparison to fluorescence microscopy, which depends on chemical labeling and is susceptible to limitations like photobleaching and cytotoxicity, PS‐HT is entirely non‐invasive and compatible with live‐cell imaging.^[^ ^33^ ^]^ This technique uniquely facilitates prolonged and perturbation‐free monitoring of LD dynamics, which is critical for investigating lipid metabolism under physiological and pathological contexts. In live‐cell experiments, minor subcellular motion between successive polarization acquisitions may occur; therefore, image alignment and tracking algorithms might be required to compensate for such displacements and ensure accurate birefringence reconstruction.

Looking ahead, the applicability of PS‐HT imaging can be extended to diverse biological models, such as stem cells, immune cells, and metabolic disorders. Its inherent compatibility with machine learning‐driven analysis further enhances its potential by enabling automated classification of cellular states based on LD‐specific parameters. Additionally, integrating PS‐HT into high‐throughput screening platforms could significantly advance drug discovery efforts targeting lipid metabolism and related pathways.

In summary, our findings establish PS‐HT as a robust, versatile, and label‐free imaging approach capable of delivering comprehensive morphological, biophysical, and structural insights into lipid droplets. Beyond advancing fundamental research in lipid metabolism and disease mechanisms, this approach holds significant potential for translational applications – from basic biomedical research to clinical diagnosis.

## 4 Experimental Section

### Cell Culture, Glc Treatment, and Confocal Microscopy

PNT2 cells (95 012 613) were obtained from the European Collection of Cell Culture (ECACC), while PC3 cells (CRL‐1435) were sourced from the American Type Culture Collection (ATCC). PNT2 cells were cultured in RPMI medium supplemented with 10% (v/v) FBS, 2 mm L‐glutamine, 100 U mL^−1^ penicillin, and 100 µg mL^−1^ streptomycin. PC3 cells were maintained in DMEM/F12 medium with the same supplements. All cell cultures were kept in a humidified incubator at 37 °C with 5% CO_2_. Seventy‐two hours prior to the experiment, PNT2 and PC3 cells were seeded in 24‐well plates on coverslips at densities of 25 000 and 50 000 cells per well, respectively. Eight hours post‐seeding, the culture medium was replaced with fresh DMEM supplemented with 5 mm deut‐Glc. The following day, this medium was substituted with a 2 mm Glc medium for 1 h. Subsequently, cells were washed three times with PBS and either maintained without Glc or cultured for 48 h in a medium containing 25 mm deut‐Glc. After the culturing time in deut‐Glc, cells were fixed for 10 min at room temperature in 4% (v/v) paraformaldehyde, and then washed three times with phosphate‐buffered saline (PBS) 1×. Cells were incubated with 2.5 µm Bodipy for LDs staining, 2 µg mL^−1^ Hoechst for nucleus staining and 33 nm Alexa Fluor 546‐phalloidin for F‐actin staining diluted in PBS 1× for 30 min at room temperature in the dark. After incubation, cells were washed three times with PBS to eliminate excess dye. Coverslips were mounted with ProLong Gold Antifade Mountant and examined by confocal microscopy (Zeiss LSM 980).

### PS‐HT and WFM Microscopy

For microscopy, the coverslips with immobilized cells were first immersed in a chamber (TomoDish) containing 20 µL of PBS. HT and PS‐HT imaging were performed using a Tomocube HT‐2H holographic microscope, which was also modified to allow the study of cellular birefringence (see Figure 1). The system also integrated WFM capabilities, allowing for multimodal imaging. The holographic part of the microscope employed a Class I diode laser light (wavelength: 532 nm, output power < 0.4 mW) with linear polarization as a light source. The laser beam was split into reference and object beams using a beam splitter. The object beam interacted with the cell at varying incident angles, controlled by a digital micromirror device (DMD), enabling tomographic imaging. To analyze the birefringent distribution, the linear polarization state of the object beam was adjusted using a half‐wave plate (Thorlabs, AHWP10M‐600). Due to the passage through the sample, the state of polarization of the beam (Obj) could change. An objective microscope (60× NA 1.2 water immersion) was used to collect the wave emitted from the sample. On the other hand, the linear polarization of the reference beam was controlled using a half‐wave plate (Thorlabs, AHWP05M‐600) mounted on a motorized mount. This setup allowed the linear polarization to be switched between two orthogonal states (Ref1 and Ref2) and to ensure that the polarization of the object beam was oriented at a 45° angle relative to these two orthogonal polarizations. The alignment of the reference beam's polarization vector relative to the object beam was crucial for optimal interference (Figure 2). The interference between the object beam (Obj) and the reference beam with polarization 1 (Ref1) or polarization 2 (Ref2) was acquired (for each incident angle) using a 2.8‐megapixel CMOS sensor with an exposure time of 0.4 seconds. The reconstructed 3D images had an axial resolution of 220 nm, a lateral resolution of 110 nm, and a depth along the *z*‐axis of 40 µm. The holograms corresponding to the vertical polarization were recorded immediately after the horizontal polarization holograms, with a minimal time interval of ≈5 s to maintain experimental consistency.

For WFM microscopy, non‐confocal sectioning was performed using an LED excitation source with three filter sets (385, 475, and 570 nm). The lateral resolution was 220 nm, while the axial resolution was ≈1 µm. Both PS‐HT and WFM imaging shared a field of view of 80 µm × 80 µm, ensuring precise spatial correlation between the two modalities. WFM imaging was conducted immediately before HT from the same position to avoid photobleaching and to maintain alignment for direct comparison of the results.

### Blind Richardson–Lucy Deconvolution Algorithm

Blind Richardson–Lucy deconvolution was applied using MATLAB's Image Processing Toolbox to process WFM images. The iterative deconvolution was performed using the “deconvlucy” function, which refined the point spread function estimation. Custom MATLAB scripts were implemented for PSF initialization and adaptive regularization. To enhance spatial resolution, the algorithm was executed with specific constraints on noise suppression and edge preservation. Convergence was assessed using normalized root mean square error metrics, and deconvolution results were validated against holotomography data.

### Optimal *RI* Threshold Calculation

The colocalization coefficients *M*_1_ and *M*_2_ are defined as follows:

(2) $$M_{1}=\left(N_{\mathrm{WFM}}\;\cap \;N_{\mathrm{HT}}\right)/N_{\mathrm{WFM}}$$

(3) $$M_{2}=\left(N_{\mathrm{WFM}}\;\cap \;N_{\mathrm{HT}}\right)/N_{\mathrm{HT}}$$

where *N*_WFM_ and *N*_HT_ are the total number of LD‐associated pixels in the binary MIP images obtained from WFM and HT, respectively (Figure 2c,d). The intensity thresholding for the green channel in WFM was consistently applied to all images to ensure uniformity in image processing. As the *RI* threshold changes, the pixel regions classified as LDs in HT also vary; consequently, different *RI* thresholds yielded different *M*_1_ and *M*_2_ values. When the *RI* threshold was set to a small value (typically below 1.36), the LD regions in HT became larger than their true size, causing *M*_1_ ≈ 1 while *M*_2_ ≪ 1. Conversely, selecting a higher *RI* threshold (typically above 1.3800) resulted in an underestimate of LD regions in HT, making *M*_1_ ≪ 1 while *M*_2_ ≈ 1. Here, computed *M*_1_ and *M*_2_ were computed for *RI* thresholds ranging from 1.36 to 1.38, and the smallest *RI* that maximized *M*_2_ was identified as the optimal *RI* threshold (*RI*_th_). Beyond this optimal value (*RI* >* RI*_th_), changes in *M*_2_ became negligible.

### Image Alignment

Since the coverslip within the TomoDish chamber was not fixed and remained submerged, it was subjected to displacement during the time interval between the acquisition of the first and second polarization images. However, given the rapid acquisition process (≈0.4 s), any sample displacement during image acquisition could be considered negligible. To achieve lateral alignment of the 3D images, the image acquired under the first polarization was designated as the reference, while the image obtained under the second polarization was treated as the moving image. To maximize the feature content for alignment, maximum intensity projection (MIP) along the depth (*z*‐axis) was generated for both polarization images. An ROI was then defined around the cell to exclude features outside the cellular structure. Subsequently, image registration was performed using the “imregister” function in MATLAB, aligning the moving image to the reference image through rigid transformations, which included rotation and translation without scaling. Both images were then cropped laterally to ensure that any displacements or rotations of the moving image introduced during the alignment process were effectively corrected. The transformation function obtained from the alignment of the MIP images was applied uniformly to all slices of the moving image, thereby achieving complete lateral alignment. In addition to lateral alignment, axial alignment (along the *z*‐axis) was necessary, as corresponding slice numbers between the two images might not be identical. Similar to the lateral alignment approach, one image was selected as the reference while the other was treated as the moving image. Five random slices were extracted from the reference image, and their corresponding slices in the moving image were identified using a mutual information‐based image registration algorithm. The average shift across these five slices was computed and rounded to the nearest integer. By applying this shift to the moving image, complete axial alignment was achieved, ensuring precise registration of the two datasets.

### Segmentation and Volumetric Analysis

The segmentation process was performed on *RI* and birefringence images using the free and open‐source software 3D Slicer. Initial segmentation on HT images was carried out by applying *RI* thresholds specific to each subcellular organelle. For birefringence images a fixed threshold of 0.01 was applied. To refine the segmentation, small islands and unrelated regions were removed by applying size filtering and defining an ROI. Subsequently, a smoothing process was applied using a Gaussian filter. The number of voxels in each segmented region was then computed, and the corresponding volume for each segmentation was calculated.

### Statistical Analysis

All quantitative data were presented as mean ± standard deviation (SD). Statistical significance was assessed in MATLAB using two‐sided Wilcoxon rank‐sum tests. Significance levels were indicated as follows: *p < 0.05, **p < 0.01, ***p < 0.001, ****p < 0.0001.

## Conflict of Interest

The authors declare no conflict of interest.

## Author Contributions

A.C.D.L. and G.C. contributed equally to this work. H.K. and M.M. contributed to the study design, execution, and data acquisition. H.K. performed the image processing. All authors contributed to data analysis and interpretation. H.K. drafted the manuscript, and all authors revised and critically reviewed the article; approved the final version to be published; agreed on the journal of submission; and accepted responsibility for all aspects of the work. A.C.D.L., M.A.F., and G.C. conceived the study, supervised the project, and secured funding.

## Supporting information



Supporting Information

Supplemental Movie 1

Supplemental Movie 2
