# Supplementary material for: Polarization‐Sensitive Holotomography for Multidimensional Label‐Free Imaging and Characterization of Lipid Droplets in Cancer Cells
Source: Adv Sci (Weinh). 2025 Aug 21;12(42):e09420. doi: 10.1002/advs.202509420 (PMC12622439; doi:10.1002/advs.202509420)
Supplement: Supplementary file 1 — Supporting Information [file ADVS-12-e09420-s002.docx]

**Supporting Information**

**Polarization-sensitive holotomography for multidimensional label-free imaging and characterization of lipid droplets in cancer cells**

*Hossein Khadem ^1^, Maria Mangini ^1,*^, Maria Antonietta Ferrara ^2,*^, Anna Chiara De Luca ^1,†^, Giuseppe Coppola ^2,†^*

*^1^ Institute of Endotypes in Oncology, Metabolism and Immunology "Gaetano Salvatore" IEOMI, Second Unit, National Research Council, Via P. Castellino 111, 80131, Naples-Italy;*

*^2^ Institute of Applied Sciences and Intelligent Systems “Eduardo Caianiello” ISASI, Unit of Naples, National Research Council, Via P. Castellino 111, 80131, Naples-Italy;*

*^*^ Correspondence to:* [*maria.mangini@cnr.it*](mailto:maria.mangini@cnr.it)*;* [*mariaantonietta.ferrara@cnr.it*](mailto:mariaantonietta.ferrara@cnr.it)

*^†^* *These authors contributed equally to this work.*

**I. Supplementary Figure**

**
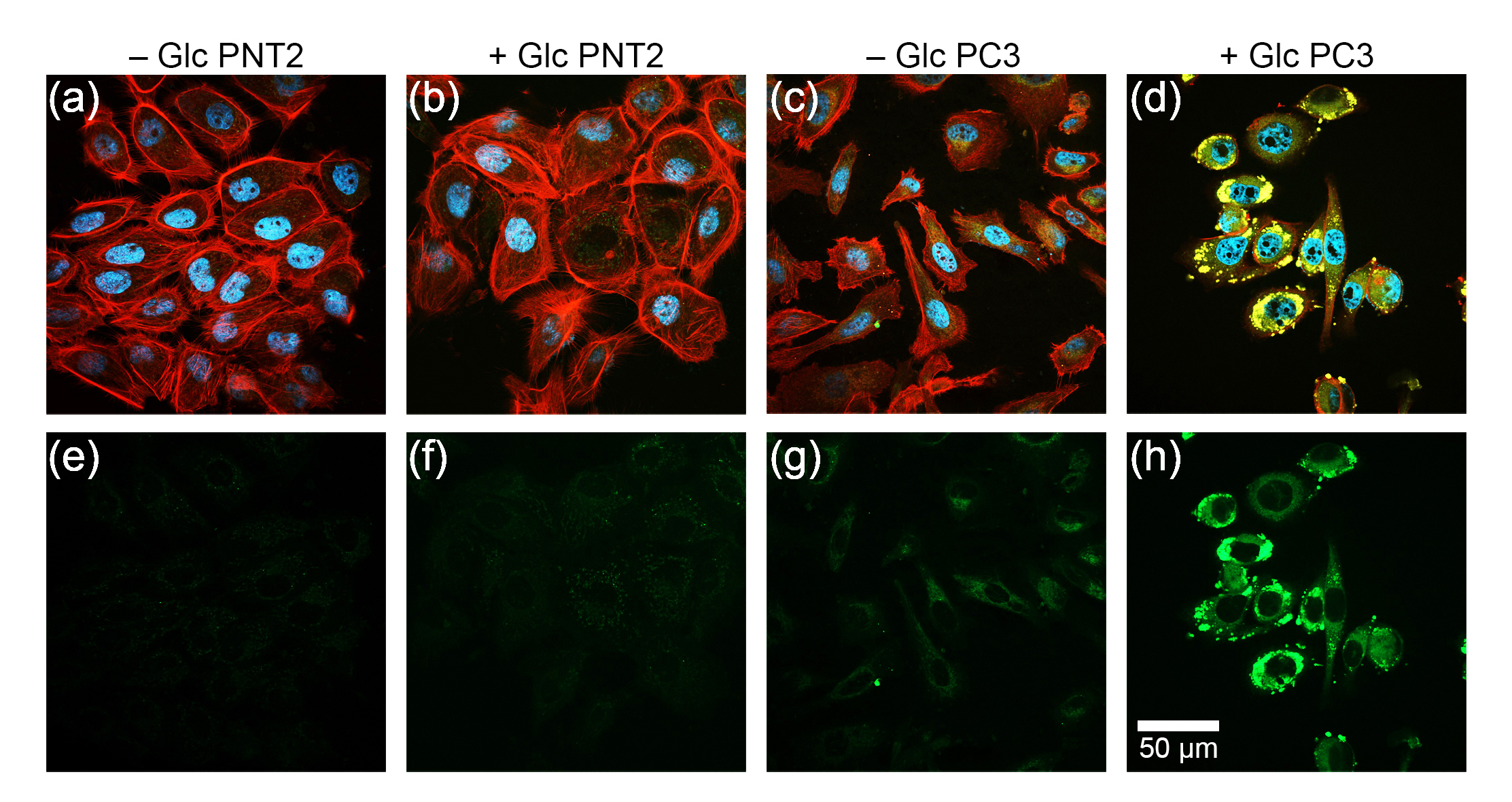
**

**Figure S1:** Confocal fluorescence microscopy (CFM) images demonstrating LD formation in cancer cells following Glc treatment. **(a–d)** Merged CFM images of PNT2 (healthy) and PC3 (cancer) cells under untreated (–Glc) and glucose-treated (+Glc) conditions. **(e–h)** Corresponding green fluorescence channel showing BODIPY-stained LDs. A qualitative comparison reveals a marked increase in LD accumulation in glucose-treated PC3 cells, characterized by larger and more abundant droplets compared to other cell lines.Glc-treated PC3 cells exhibit significantly higher LD content relative to both untreated PC3 cells and all PNT2 conditions.


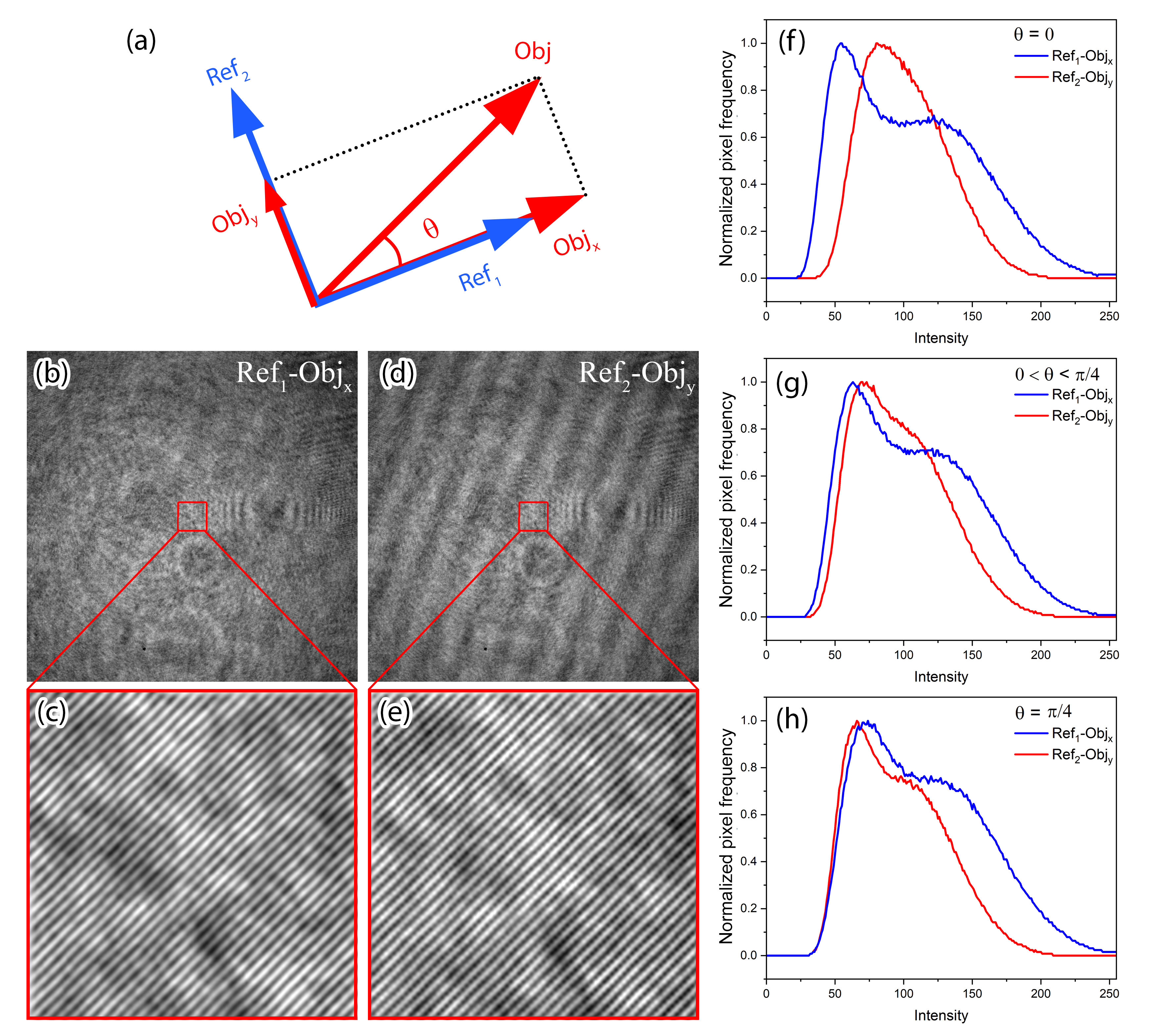


**Figure S2: (a)** Orientation of the polarization vectors of the object (Obj) and reference (Ref) beams. The angle *θ* represents the angle between the Obj and its projected component onto the Ref_1_ polarization vector (Obj_x_). The component of the Obj vector projected onto the Ref_2_ polarization vector is denoted as Obj_y_. By rotating the half-wave plate (HWP), the directions of Ref_1_ and Ref_2_ vectors are adjusted so that *θ* is set to 45 degrees. **(b, c)** The interference pattern resulting from the interaction of Ref_1_ and Obj_x_ and its magnified view, respectively. **(d, e)** The interference pattern resulting from the interaction of Ref_2_ and Obj_y_ and its magnified view, respectively. **(f–h)** Histograms of the interference patterns for *θ* values of 0 degrees, between 0 and 45 degrees, and 45 degrees, respectively. **HWP adjustment:** As observed, for *θ* = 0 degrees, the interference pattern consists of alternating dark (peak with intensity ~50) and bright (peak with intensity ~150) fringes only for the interaction between Ref_1_ and Obj_x_. The interaction between Ref_2_ and Obj_y_ results in destructive interference, forming a single gray peak with an intensity of ~100. As *θ* increases, an interference pattern appears for both interactions. When *θ* reaches 45 degrees, both interactions exhibit interference patterns with similar pixel frequencies.

**
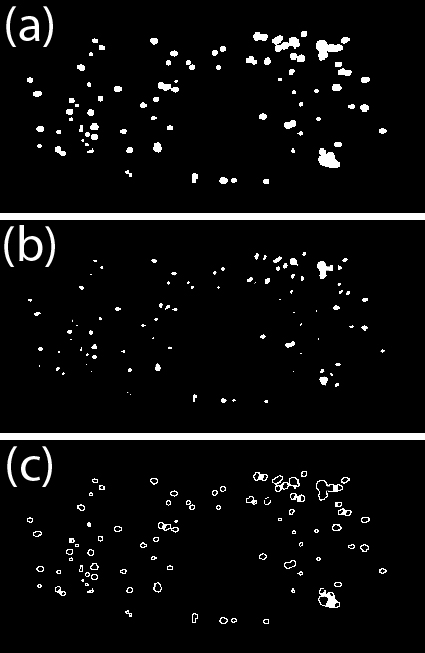
**

**Figure S3:** Binary images of LDs obtained using **(a)** WFM and **(b)** HT of a Glc-treated PC3 cell as shown in Fig. 2c,d. **(c)** Absolute difference between WFM and HT images. Despite the high degree of overlap between the two images, LDs appear larger in the WFM image due to different optical resolution and point spread functions of the methods. This effect results in generally higher M_2_ colocalization coefficients compared to M_1_ (Fig. 2e-h).

**
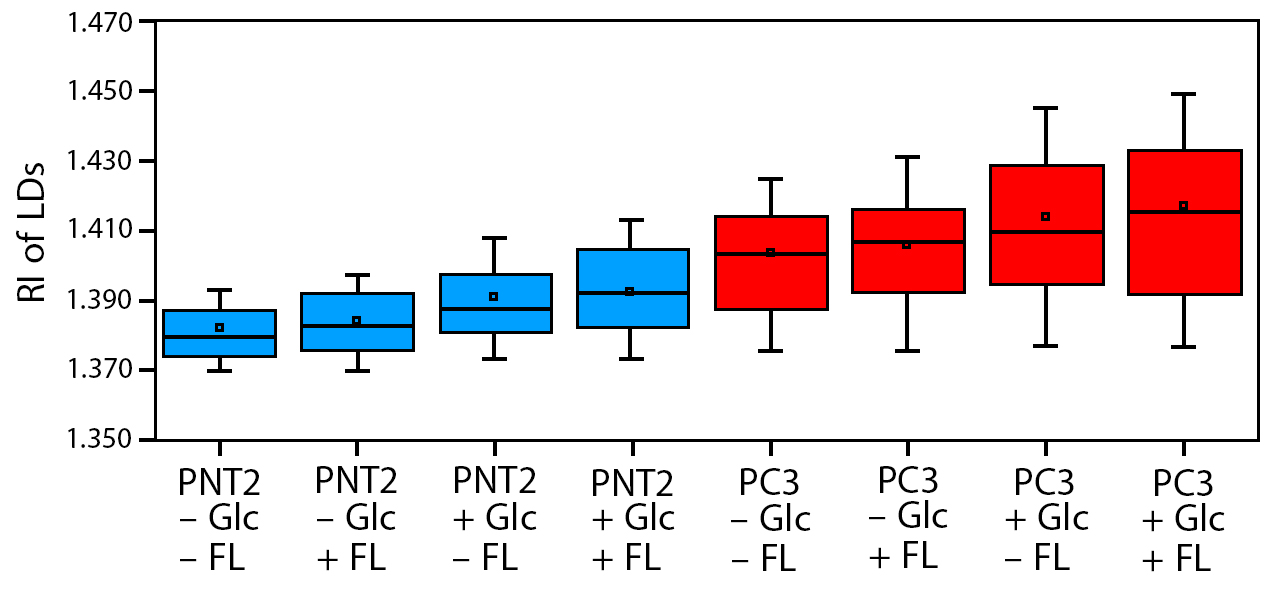
**

**Figure S4:** Comparison of the RI of LDs in PNT2 and PC3 cell lines, both untreated (-Glc) and treated with glucose (+Glc), under two conditions: unlabeled (-FL) and labeled with BODIPY fluorescence (+FL). This graph presents the distribution of the RI of voxels that exceed a defined threshold (RI_th_ obtained from colocalization analyses) and are located in the cytosolic region. As observed, fluorescence labeling slightly increases the RI of LDs; however, the change in their mean RI is negligible. Therefore, the effect of fluorescence labeling on the RI of LDs can be disregarded. It is important to note that unlabeled and labeled cells are different populations. If experimental conditions are designed to measure RI changes due to labeling within the same cells, the results would be more precise, and the variations in RI would likely be smaller.

**
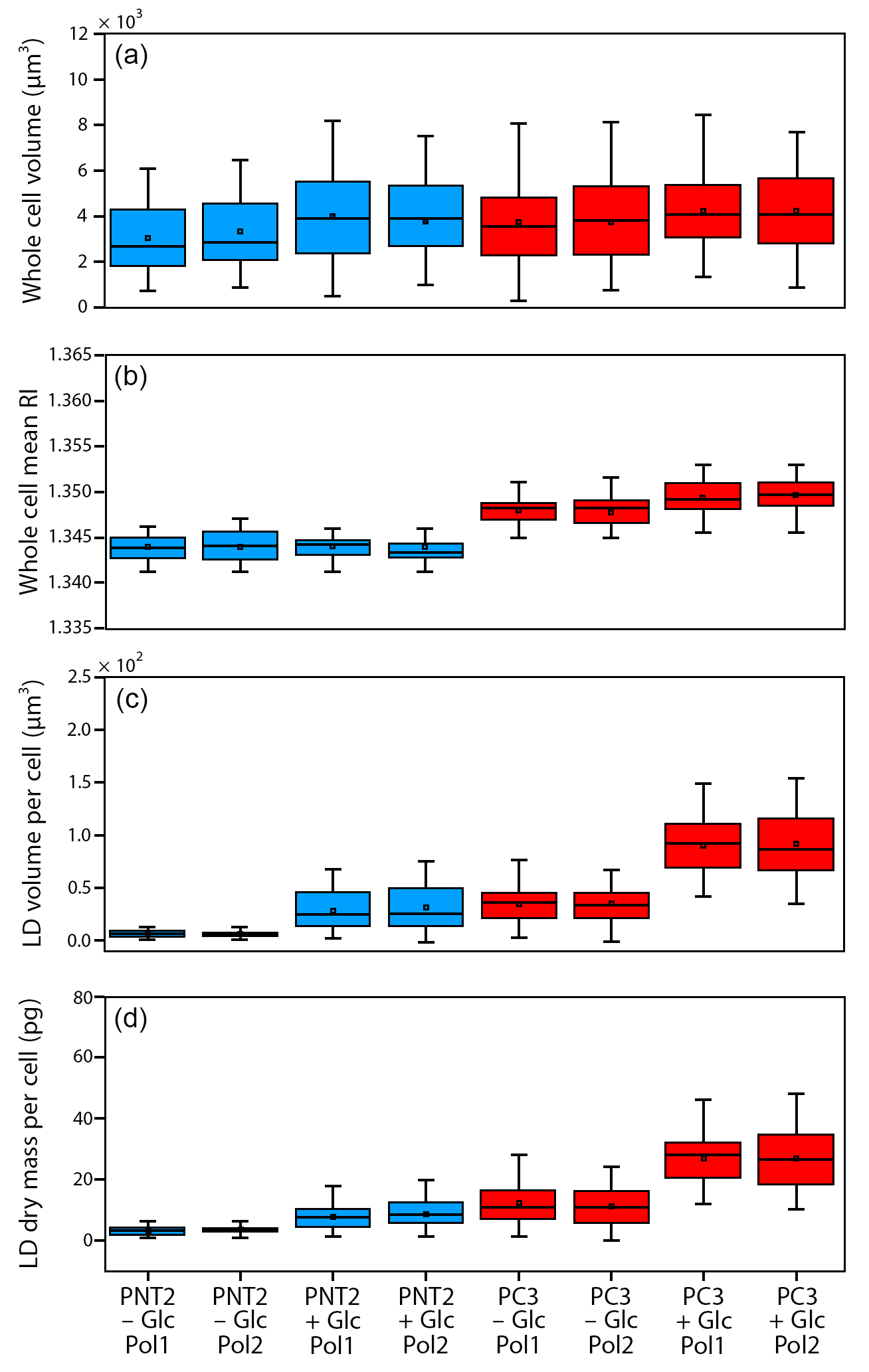
**

**Figure S5:** Quantitative results of cellular physical parameters measured using HT for two orthogonal polarizations, Pol1 (vertical) and Pol2 (horizontal). Although some differences can be observed in the quartiles and medians between polarization states, the mean value—indicated by the square marker—remains nearly unchanged. Since the same cells were analyzed for both Pol1 and Pol2, the results are very similar, indicating that polarization has minimal impact on the quantitative measurements.To compare the refractive index difference between orthogonal polarization states in **(b)**, we define Δ*RI* = $\left| {RI}^{↕}-{RI}^{\leftrightarrow} \right|$ and the results for mean values (square markers) are: Δ*RI*(PNT2 −Glc) = 0.0000, Δ*RI*(PNT2 +Glc) = 0.0000, Δ*RI*(PC3 −Glc) = 0.0002, and Δ*RI*(PC3 +Glc) = 0.0003. The concentration (and consequently, the dry mass in **(d)**) is calculated using Barer’s relation, given by: $\text{C}\text{ }\text{=}\text{ }\text{(}\text{n}\text{ }\text{-}{\text{ }\text{n}}_{\text{0}}\text{)/}\text{α}$, where *n* is the refractive index of a voxel, *n_0_*​ is the refractive index of the surrounding medium (which is PBS in this study, with *n_0_*​=1.335), and *α* is the refractive index increment (RII), typically taken as 0.135 fL/pg for lipids. Although the anisotropic structure of LDs causes polarization-dependent variations in the RI across representative voxels, the total dry mass of LDs in a cell—calculated by integrating all associated voxels—shows minimal dependence on the polarization state. As illustrated in panel (d), this leads to negligible differences in the overall dry mass measurements between orthogonal polarizations.

**
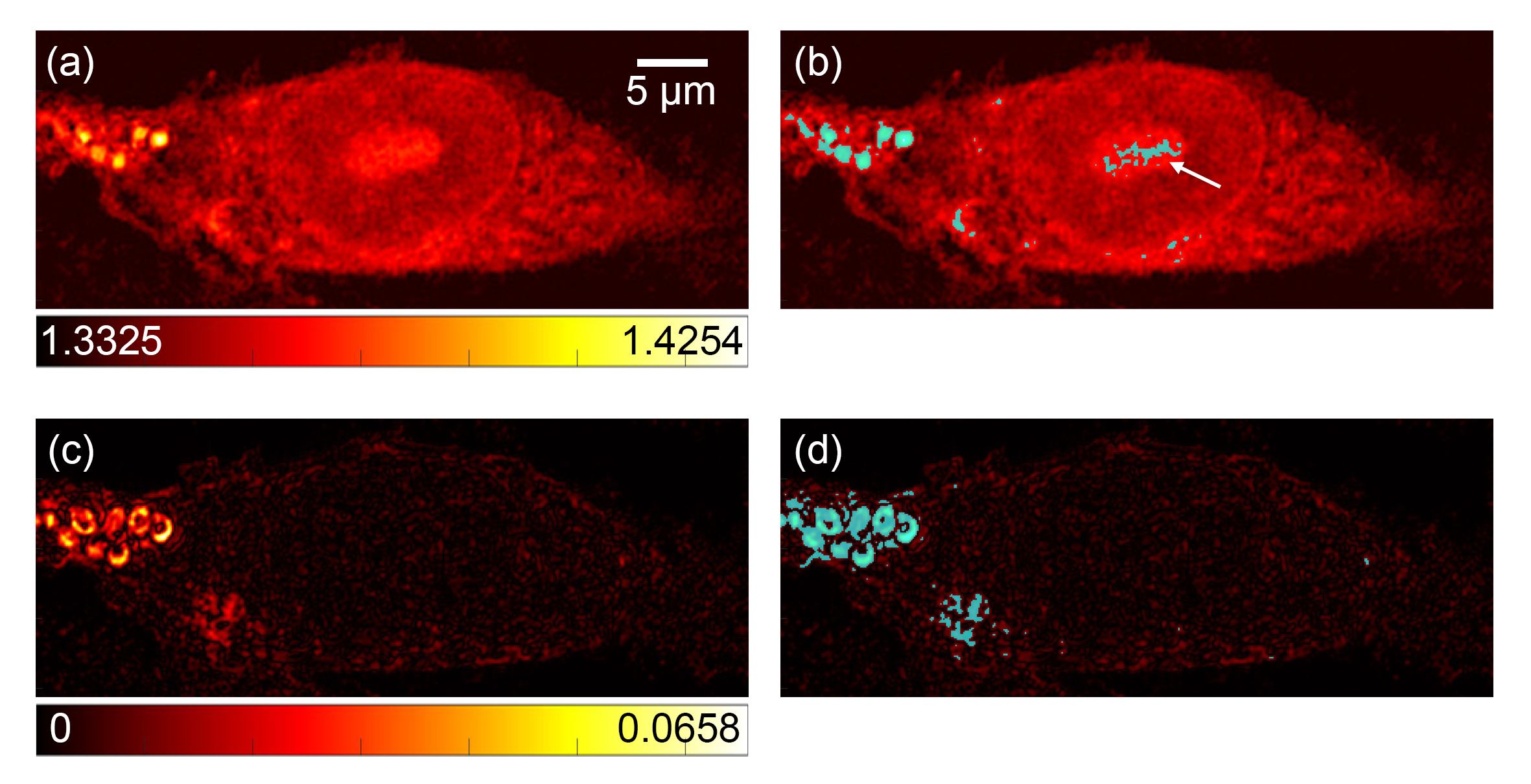
**

**Figure S6:** **(a)** RI map of a Glc-treated PC3 cell, corresponding to slice number 72 out of 152 slices. **(b)** The same image as in **(a)**, with regions having an RI greater than 1.3768 segmented in cyan. **(c)** Birefringence map corresponding to the RI map shown in **(a)**. **(d)** The same image as in **(c)**, with regions exhibiting birefringence values greater than 0.01 segmented in cyan.

Despite the ability of the RI threshold to effectively identify LDs, certain intracellular regions, such as the nucleolus, also exhibit RI values within the same range as LDs. This overlap reduces the selectivity of RI thresholding for LD identification. In contrast, in the birefringence image, only LD regions exhibit high birefringence, ensuring superior selectivity for their detection.

**
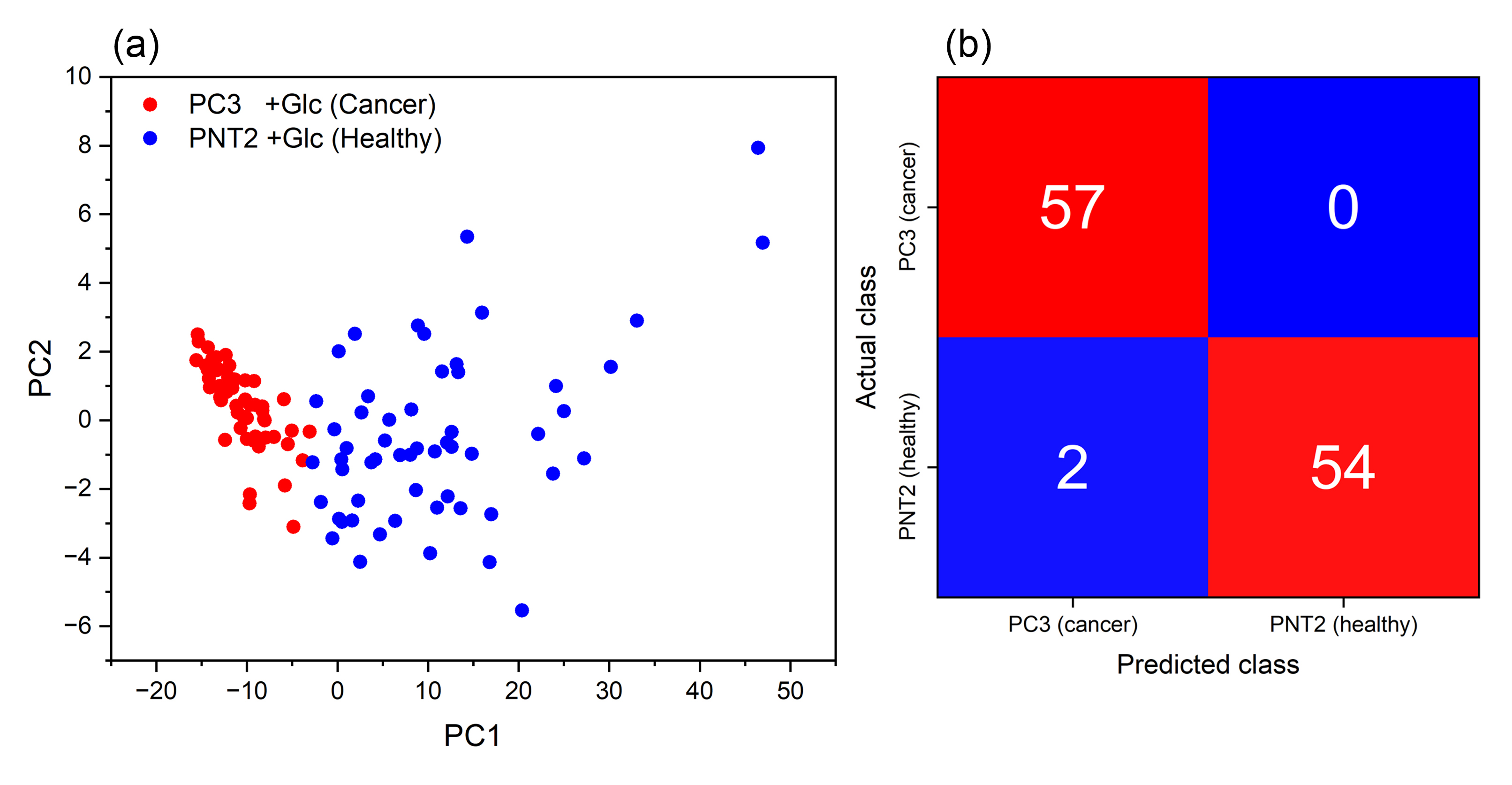
**

**Figure S7:** **(a)** Principal component analysis (PCA) score plot differentiating PC3 (red) and PNT2 (blue) cells treated with glucose (+Glc). The PCA was performed directly on the 3D birefringence images without applying any threshold. **(b)** The corresponding confusion matrix indicates the classification performance, achieving sensitivity = 100%, specificity = 96.4%, accuracy = 98.2%, and precision = 96.6%.

**II. Supplementary Table**

**Table S1:** RI range for various subcellular components. Given the importance of LDs in this study, their optimal RI threshold was determined based on maximum colocalization between HT and WFM microcopy. For other organelles, the RI range was estimated through HT image analysis. In each cell line, a region of interest (ROI) was selected based on the morphological characteristics of each organelle in HT images. The minimum and maximum RI values within this region were defined as the RI range for the respective organelle. For segmentation, both the obtained RI range and the selected ROI were applied to prevent overlap between segmented regions. Since the reported refractive index values represent the average across different cells, there is no overlap between the refractive index ranges. However, within a single cell, overlaps may exist, potentially reducing selectivity (see Figure 5).

|  | PNT2  (healthy) | | | | PC3  (cancer) | | | |
| --- | --- | --- | --- | --- | --- | --- | --- | --- |
|  | –Glc | | +Glc | | –Glc | | +Glc | |
|  | RI_min_ | RI_max_ | RI_min_ | RI_max_ | RI_min_ | RI_max_ | RI_min_ | RI_max_ |
| Nucleolus | 1.3557 | 1.3627 | 1.3546 | 1.3655 | 1.3526 | 1.3683 | 1.3614 | 1.3744 |
| Nucleus | 1.3453 | 1.3517 | 1.3494 | 1.3557 | 1.3446 | 1.3502 | 1.3551 | 1.3622 |
| LDs | 1.3698 | ∞ | 1.3732 | ∞ | 1.3754 | ∞ | 1.3768 | ∞ |
| Cytosol | 1.3391 | 1.3475 | 1.3391 | 1.3463 | 1.3406 | 1.3528 | 1.3436 | 1.3592 |
